# Practitioners' Bookshelf

**Published:** 1892-03-12

**Authors:** 


### PRACTITIONERS' BOOKSHELF.

### DISEASES OF THE OVARIES AND FALLOPIAN TUBES.*

In this book we have a work of marked originality and very great value. It is evidently the outcome of much laborious and very careful investigation, and makes a valuable addition to our knowledge of the subject. It is very clearly written, and everywhere just to the point. The pictures are exceedingly good and most abundant, a point of no small importance in a work of this character. Indeed, we can hardly recall to mind another surgical work so well illustrated. Some of the coloured plates are very fine, and all the drawings are remarkably clear. As a pathological text book it is particulary good.

The book is divided into four parts, the first is devoted to diseases of the ovary, the second to diseases of the fallopian tubes, the third to tubal pregnancy, and the fourth to the methods of performing operations for ovarian and tubal disease.

The work begins with a chapter on sex and menstruation.

* "Surgical diseases of the ovaries and Fallopian tubes including tubal pregnancy," by Bland Sutton, F.R.C.S., illustrated, pp. 488, 1891. (Cassell and Co., London.)

The author shows, as the result of numerous observations, that there is no shedding of the mucous membrane of the uterus during menstruation as is commonly taught.

On dermoid cysts of the ovary he is—as we might expect from his previous book on dermoids—particularly good; indeed, he is quite the authority on these growths. The drawings of dermoids are a particularly interesting series.

He holds strongly the view that dermoids found growing on the omentum or mesentery have been originally ovarian. He says they contract adhesions to the mesentery, and then their attachment to the ovary gets drawn out and thinned, and eventually gives way, and he tells us he has seen these cysts attached in this way to both.

Dermoid cysts of the ovary are usually regarded as quite innocent growths, and to this Bland Sutton would agree with regard to the great majority of such growths, but he quotes Thornton to the effect that in some cases where sarcoma tissue was found in ovarian dermoids, solid sarcomata recurred in the pelvis after their removal. In another case of Jessop's, the cyst contained carcinomatous growth, and there were secondary growths in the liver. We must not, he tells us, however, too readily decide that there is sarcoma tissue in these cysts, as so many tissues are represented in them.

He mentions the great length to which the hair grows in some of these dermoids, and alludes to a specimen now in the Museum of the Bristol Children's Hospital, in which is a lock of hair 28 in. in length. This cyst was removed by Mr. Ewens from a child seven years of age.

The pathology of ovarian and broad ligament cysts is well described on an anatomical basis, and the various forms clearly differentiated.

It is, of course, well known that cyst arising in the hilum of the ovary, where remains of the Wolffian body are to be found very often contains papillomatous growth, and that ordinary multilocular ovarian cysts do not. Bland Sutton describes, however, small cysts in the ovary containing warty growths, but these, he says, never attain any size, and the warty growths in them are of almost cartilagenous hardness.

There is an interesting drawing of a parovarian cyst which had undergone axial rotation and complete detachment.

In the chapter on "Ovarian Tumours in Children," he says "the malignant tumours in the ovaries of children—termed by some sarcomata, by others carcinomata—are histologically distinct from the common forms of cancer or sarcoma, and they ought to be arranged provisionally in a group by themselves under the term oöphoromata, because they seem to be special to the connective tissue of the oöphoron," and by this he means the ovary as distinct from the hilum. Then he gives us the distinguishing characters of these oöphoromata; but they do not seem to us to justify their being placed in a separate class to sarcomata and carcinomata. These reasons are as follows:

1. Histologically they repeat the characters of the connective tissue of the fœtal ovary.

2. It usually affects both ovaries.

3. They rarely occur after puberty.

4. Sometimes they occur in association with ovarian dermoids before puberty, and lead to secondary deposits.

5. They recur locally after removal.

6. In fœtuses and very young children the cell elements preponderate.

7. Towards puberty they tend to assume an alveolar arrangement and mimic the structure of cancer.

Now as Bland Sutton himself points out it is a remarkable character of solid primary sarcomatous growths of the overy to affect both at the same time. Fœtal sarcomata would probably resemble fœtal connective tissue. That they occur before puberty is presupposed. Sarcomatous growth has been described by Thornton associated with dermoids. All sarcomata tend to recur locally. The preponderance of cell element, and the presence of alveolar arrangement, surely do not remove them from the same class as the sarcomatous or carcinomatous growths. Bland Sutton may have seen histological peculiarities which would separate them, but these reasons seem quite insufficient to do so.

But we must not give the whole of the space at our disposal to ovarian growths, but will very briefly allude to some other subjects.

Ovarian apoplexy is defined, as hæmorrhage into the ovarian stroma through rupture of a follicle. In rare cases the ovary may thus become distended with blood, or may rupture and lead to an hæmatocele in Douglas' pouch. But this is very rare, and cases described as such are really often ruptured tubal pregnancies. Sutton says he once reported a case of pelvic hæmatocle as being due to bursting of an enlarged ovarian follicle, which re-examination of the specimen showed to be due to a ruptured tubal gestation.

The pathology, diagnosis, and treatment of tubal pregnancy is very fully given in a series of chapters; and the practical details of ovarian operations, and the differential diagnosis of ovarian growths fully discussed.

To the gynæcologist the book must be of the greatest importance, and to the abdominal surgeon invaluable. We are sorry to see a tendency at the present day to separate pathology from practice, but surely sound rational treatment depends much on pathological knowledge. What is pathology but a knowledge of the thing that we have to deal with as operating surgeons or gynæcologists? It is not some collateral science relating to it as too often we might suppose from its dissociation from diagnosis and treatment. It is by careful and thorough work in the post-mortem room and examination of morbid growths removed during life that we shall gain a knowledge of disease of immense practical value when we have to diagnose and to operate, and of no department of work is this more true than in ovarian diseases. This is why a work of this kind—a work with a sound pathological basis—is so valuable also as a practical guide.

